# Cell division during Xenopus gastrulation influences neuroectoderm patterning

**DOI:** 10.3389/fcell.2026.1798565

**Published:** 2026-04-22

**Authors:** Ian Velloso, Rodrigo Araujo, Marko Horb, Jose G. Abreu

**Affiliations:** 1 Instituto de Ciências Biomédicas, Universidade Federal do Rio de Janeiro, Cidade Universitaria, Rio de Janeiro, Brazil; 2 Eugene Bell Center for Regenerative Biology and Tissue Engineering and National Xenopus Resource, Marine Biological Laboratory, Woods Hole, MA, United States

**Keywords:** A-P patterning, brain malformation, cell division, mitosis, neural plate, neural tube anomalies, *Xenopus laevis*

## Abstract

Oriented cell division is emerging as a fundamental morphogenetic mechanism driving tissue elongation and axis formation. Although *Xenopus laevis* embryos can undergo gastrulation and neurulation in the absence of cell division, the specific contribution of mitosis during gastrulation remains poorly defined. Here, we systematically examine the role of cell division during gastrulation in neural plate formation and anterior–posterior (A–P) patterning. Using Hydroxyurea and Aphidicolin (HUA) to block cell division, combined with in situ hybridization and time‐lapse imaging, we analyzed division dynamics and developmental outcomes in the dorsal mesoderm and neuroectoderm. Consistent with previous work, we find that cell division is dispensable for dorsal mesoderm patterning and neural tube closure. Strikingly, however, inhibition of cell division during gastrulation profoundly disrupts neural plate patterning, leading to severe head and trunk defects that manifest at tailbud stages. This phenotype is accompanied by markedly reduced expression of key anterior neural markers (*bf1 and krox20*), indicating a failure to properly specify the forebrain, midbrain, and hindbrain domains. These defects reveal an essential role for cell division in early neural regionalization rather than in tissue formation per se. Moreover, quantitative analysis shows that cell divisions are abundant throughout the gastrulating ectoderm and exhibit strong A–P orientation, particularly in the dorsal posterior region. Together, our results identify A–P oriented cell division as a conserved and previously underappreciated mechanism required for neural plate elongation and anterior neural patterning during vertebrate development.

## Graphical Abstract

**Figure:**
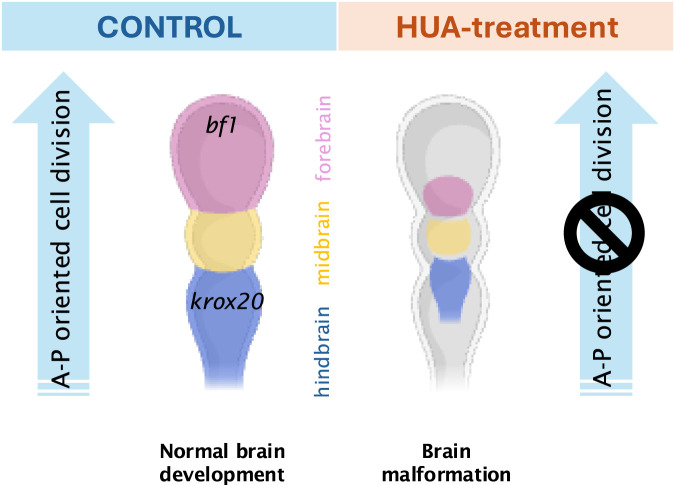

## 1 Introduction

The matter of how animals assume their definitive shape has been fascinating biologists for centuries. Amphibian’s early embryogenesis is particularly generous in showing the steps by which a round embryo is transformed into a bilaterian elongated body, typical of vertebrates. A process which includes formation and patterning of the notochord, somites, neural plate/tube, and spinal cord (Gilbert and Barresi, 2016; Sive et al., 2000). In this context, the concept of neural induction has been extensively explored. And the cellular behaviors responsible for neural structures to be formed, aligned with the A-P body axis, vary among vertebrates. In amniotes, for example, convergent extension (CE) is responsible for the elongation of the prospective brain (forebrain, midbrain, and hindbrain) (Hatada and Stern, 1994). Whereas the spinal cord is formed through posterior growth, as bipotent neural mesodermal progenitor (NMp) cells proliferate and are allocated either towards dorsal mesoderm or spinal cord (Le Douarin and Halpern, 2000; Charrier et al., 1999; Selleck and Stern, 1991; Brown and Storey, 2000). In amphibians, on the other hand, the whole neural plate is thought to elongate due to convergent extension (CE) of the dorsal marginal zone and the spinal cord is mainly derived from the posterior portion of the neural plate (Sive et al., 2000; Keller et al., 1992a; Keller et al., 1992b) Except for the floorplate, which was shown to assemble through allocation of bipotent cells that lie within the dorsal blastopore lip (López et al., 2003; López et al., 2005). Importantly, however, there is no evidence of proliferating activity of those bipotent cells during gastrulation or neurulation (read Steventon and Arias 2017 for details regarding different mechanisms of spinal cord formation among vertebrates) (Steventon and Arias, 2017).

The convergent extension phenomenon has been very much explored in *Xenopus laevis* early embryogenesis. It has been established that medio-lateral intercalation, coupled with radial intercalation, of cells composing the dorsal marginal zone (DMZ) of the embryo is a key driving force for CE to take place (Keller et al., 1992b; Keller, 1985). At the ventral marginal zone (VMZ), on the other hand, medio-lateral intercalation, uncoupled from radial intercalation, results in thickening, rather than lengthening, of the tissue (Keller and Danilchik, 1988). It has also been proposed that cell division might be important in defining the neural plate territory during gastrulation in *Xenopus laevis* (Keller, 1978), and indeed, cell division occurs with high frequency at the epithelial neuroectoderm cells of the gastrulating *Xenopus laevis* (Chartrain et al., 2011; Hatte et al., 2014; Saka and Smith, 2001). Accordingly, oriented cell division significantly contributes to the establishment of the anterior-posterior body axis in various organisms, including insects, zebrafish, mice, and chick (Sander, 1976; Concha and Adams, 1998; Keller, 2006; Gong et al., 2004; Schoenwolf and Alvarez, 1989; Schoenwolf and Alvarez, 1992; Schoenwolf and Yuan, 1995; Wei and Mikawa, 2000).

It has been demonstrated that *Xenopus laevis* late gastrulation and neurulation occur in the absence of cell division (Harris and Hartenstein, 1991; Pokrovsky et al., 2021). However, the embryo does not continue developing if cell division is inhibited from the late blastula stage onward (Harris and Hartenstein, 1991), which suggests that cell division may be essential for the initial stages of gastrulation. Moreover, it has been shown that blocking cell division during gastrulation and neurulation affects epigenetic control as it leads to abnormal histone modification profiles (Pokrovsky et al., 2021). In this study, we have undertaken the challenge of examining the role of cell division during the morphogenesis of the neural plate and dorsal mesoderm, with particular emphasis on the early steps of gastrulation. An earlier version of this work has been deposited in bioRxiv (Velloso et al., 2025).

## 2 Materials and methods

### 2.1 Obtaining embryos

Adult frogs (Nasco Inc., WI, United States or NXR, MBL, MA, United States) were stimulated with human chorionic gonadotropin (HCG-Sigma). *Xenopus laevis* embryos were obtained by *in vitro* fertilization, dejelied with cysteine solution 2% (diluted in Barth 0,1X; pH 7.8) and cultured in Barth 0,1X (Barth 10x: 880 mM NaCl; 10 mM KCl; 10 mM MgSO4; 50 mM HEPES (pH 7.8); 25 mM NaHCO3; Kanamycin (0.1 g). The embryos were staged according to Nieuwkoop and Faber (1994) (Nieuwkoop and Faber, 1994). At NXR, MBL we used wild type frogs (NXR_0031) and the transgenic lineage NXR_0139 Xla.Tg (CMV:hist2h2be-GFP; CMV:mRFP).

### 2.2 Blocking cell division

**FIGURE 1:**
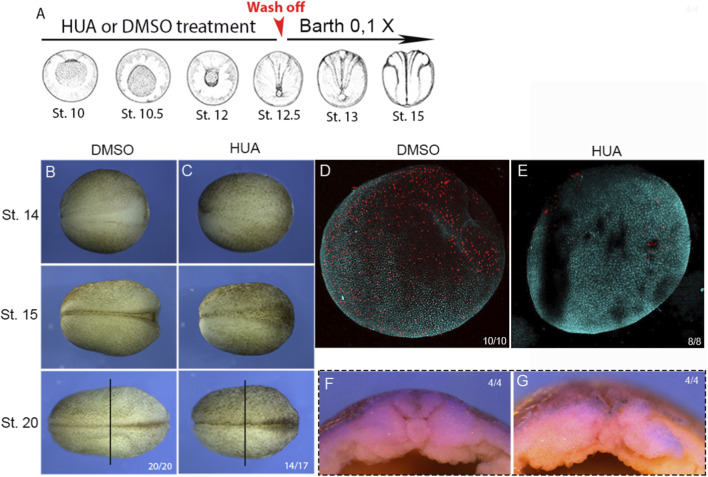
Blocking cell division during gastrulation. **(A)** Embryos were treated with a combination of Hydroxyurea and Aphidicolin (HUA) or DMSO from stage 9.5 to stage 12.5 and then transferred to Barth’s 0.1X solution starting at stage 12.5. Wash off moment is indicated in red (sages illustrated according to Nieuwkoop and Faber, 1994). **(B)** Dorsal view of a representative embryo treated with DMSO, showing the neural tube closure steps from stage 14 to stage 20. **(C)** Dorsal view of a representative embryo treated with HUA, showing the neural tube closure steps from stage 14 to stage 20. 14 out of 17 HUA-treated embryos showed the phenotype presented in C (embryos in B and C placed anterior right and posterior left). Three embryos showed a normal phenotype, similar to all DMSO-treated embryos **(D)** Dorsal view of an embryo treated with DMSO at stage 13 and immunostained for Histone H2B, showing cells in division. **(E)** Dorsal view of an embryo treated with HUA at stage 13 and immunostained for Histone H2B. **(F)** Transversal section of a DMSO-treated embryo at stage 20, showing notochord, paraxial mesoderm, and neural tube. **(G)** Transversal section of an HUA-treated embryo at stage 20, showing notochord, paraxial mesoderm, and neural tube. The vertical black line in A and B indicates the site where the transversal section was made.

**FIGURE 2:**
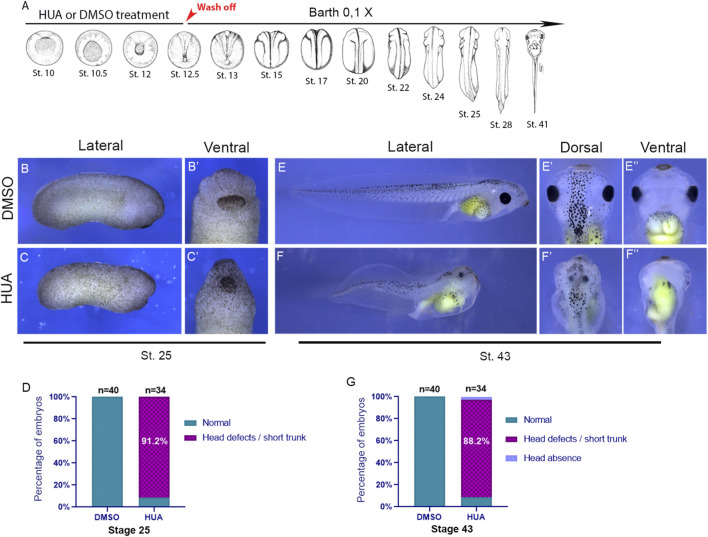
Blocking cell division during gastrulation leads to head and trunk defects. **(A)** Embryos were treated with a combination of Hydroxyurea and Aphidicolin (HUA) or DMSO from stage 9.5 to stage 12.5 and then transferred to Barth’s 0.1X solution starting at stage 12.5 (stages illustrated according to Nieuwkoop and Faber, 1994) **(B)** Lateral view of a representative DMSO-treated embryo at stage 25. Wash off moment is indicated in red. **(B′)** Ventral-anterior view of a representative DMSO-treated embryo at stage 25. **(C)** Lateral view of a representative DMSO-treated embryo at stage 25. **(C′)** Ventral-anterior view of a representative HUA-treated embryo at stage 25. **(D)** Phenotype quantification at stage 25. **(E)** Lateral view of a representative DMSO-treated embryo at stage 43. **(E′)** Dorsal-anterior view of a representative DMSO-treated embryo at stage 43. **(E′′)** Ventral-anterior view of a representative DMSO-treated embryo at stage 43. **(F)** Lateral view of a representative HUA-treated embryo at stage 43. **(F′)** Dorsal-anterior view of a representative HUA-treated embryo at stage 43. **(F′′)** Ventral-anterior view of a representative HUA-treated embryo at stage 43. **(G)** Phenotype quantification at stage 43.

Cell division was blocked by treatment with the drugs Hydroxyurea and Aphidicolin (HUA). Hydroxyurea inhibits DNA synthesis by blocking ribonucleotide diphosphate reductase, an enzyme that catalyzes the conversion of ribonucleotides into deoxyribonucleotides. This is an essential step in DNA biosynthesis. The drug Aphidicolin blocks eukaryotic DNA polymerase alpha. Aphidicolin stock solution was diluted in DMSO at a concentration of 10 mg/mL (30 mM) and frozen at −20 °C in several aliquots. On the day of treatment, the aliquot was thawed and diluted in 0.1X Barth solution to reach a working concentration of 150 uM Aphidicolin (0.5% of aphidicolin diluted in DMSO). Hydroxyurea was diluted at 20 mM. The control solution was 0.5% DMSO in 0.1X Barth solution (the same concentration as the DMSO diluted Aphidicolin). The embryos used in the experiment were immersed in the HUA or DMSO solution at three different windows of development: 1. From stage 9.5 to stage 12.5 (Figure 1A); 2. From stage 13 to stage 25 (Figure 2A); or from stage 9.5 to stage 11.5 (Supplementary Figure S1). The concentrations used have already been proven effective in two separate publications, and block cell division after 2-3 h after the beginning of the treatment (Harris and Hartenstein, 1991; Pokrovsky et al., 2021). Embryos were kept at 16 °C so they took approximately 2 h to go from stage 9.5 to stage 10.

### 2.3 *In situ* hybridization

Whole embryos were fixed in MEMFA 1X (10% de MEM 10X (Mops 1 M em pH 7.4, EGTA 20 mM, MgSO4 10 mM) + 10% formaldehyde 37% + 80% Distilled water) for 2 h at room temperature or at 4 °C overnight, and then dehydrated in an ethanol series. Whole-mount *in situ* hybridization was performed according to Abreu et al. (2002) , with modifications suggested by Reversade and De Robertis (2005). After *in situ* hybridization, embryos were treated with bleaching solution (2.5% 20 × SSC, 5% formamide, 4% H_2_O_2_ in H_2_O) and photographed with the digital camera Leica DFC290 HD coupled to the microscope MZ125 (Leica).

### 2.4 Fluorescence immunocytochemistry

Whole embryos were fixed in MEMFA 1X (10% de MEM 10X (Mops 1 M em pH 7.4, EGTA 20 mM, MgSO4 10 mM) + 10% formaldehyde 37% + 80% Distilled water) for 2 h at room temperature or at 4 °C overnight, and then dehydrated in ethanol series. Whole-mount fluorescence immunocytochemistry was performed according to Lee et al. (2008), using the primary antibody Phospho-Histone H3 (Ser10) (6G3) Mouse mAb (Cell Signaling 9706) and the secondary antibody Alexa Fluor 546 goat anti-mouse IgG (REF A11003). After immunocytochemistry, embryos were imaged at the stereoscope microscope MZ10F (Leica).

### 2.5 Nucleus-GFP time-lapse imaging

To perform the Time-lapse at a cellular level, the Zeiss light-sheet 7 microscope, installed in the Central Microscopy Facility at Marine Biological Laboratory, Woods Hole, United states, was used. The microscope was programmed to take a complete image of the embryo or explant every 3 min for a total period of 10 h. The nucleus-GFP embryos were obtained from the transgenic lineage NXR_0139 Xla.Tg (CMV:hist2h2be-GFP; CMV:mRFP) were excited with a laser emitting a wavelength of 488 nm. The embryos were mounted inside a plastic tube so that it could be rotated 120°, and thus the entire extension of the embryo’s DMZ could be photographed every 3 min. The acquisitions were performed with ×20 objectives and with a 3 um increment between each photographed plane. Importantly, the distinct focus planes are capable of capturing different heights of the ectoderm, but deep layers are not captured.

To quantify all cell divisions, we divided each of the time-lapses into four quadrants (Supplementary Figure S2). Thus, generating twelve distinct quadrants. Each of the quadrants was analyzed frame by frame (10 h of recording captured by 200 frames, with 3 min increment). It was possible to detect a morphological pattern typical of cells inside the cell division cycle: Chromosomes aligning at the cell equator (i.e., metaphase), and then gradual separation of the chromosomes (i.e., anaphase–telophase). Cell intercalation events were also detected but could be easily distinguished from cell division because it shows a different pattern: Gradual increase in nuclear signal as the cell emerge to the surface of the embryo, and no change in nuclear morphology (Supplementary Figure S2 and Supplementary Videos S2, S3).

To quantify the orientation of cell divisions we measured the angle of each cell division in the left-ventral area (n = 1,620), in the dorsal area (n = 2,427) and in the right-ventral area (n = 2034) using Fiji. All the measures were used to generate rose plots, and three important parameters were calculated: mean angle, circular standard deviation and V-score. The V-score is also called signed polarity index and quantifies both the strength and direction of alignment relative to the predefined axis (A-P), ranging from −1 (perfect anti-alignment) to +1 (perfect alignment), with 0 indicating random orientation (Giese et al., 2025). Strength of alignment describes how consistently the cell divisions are oriented toward a specific direction (i.e. A-P axis). The significance of orientation against the expected 90° (A-P) direction was tested using the V-test (Bats and chelet, 1981). For pairwise comparisons of polarity strength between embryonic regions (i.e., dorsal vs. left-ventral, dorsal vs. right-ventral), two-tailed Welch’s t-test was performed directly on cosine projection values. Statistical significance was determined after applying a Bonferroni correction to account for the three pairwise comparisons. All analyses were implemented in Python using NumPy and SciPy.

### 2.6 Keller explant

All microsurgical procedures were performed using a magnifying stereo microscope (Leica S8APO) and the following tools, manufactured by the experimenter: hair-looping and hair knife. The Keller explant is a technique widely used in embryological studies that aims to study cell movements involved in the gastrulation of *Xenopus laevis* (Doniach et al., 1992; Keller and Tibbetts, 1989; Wilson and Keller, 1991). It is the culture of the dorsal marginal region of an embryo adjacent to the dorsal marginal region of another embryo. Both explanted at the early stage of gastrulation (st. 10) and connected as a sandwich that preserves the deep cells inside and the epithelial cells outside. The explant should be composed of the involuting marginal zone (IMZ): deep cells destined to become notochord, somites, and also an endodermal epithelial layer; and the non-involuting marginal zone (NIMZ): ectodermal cells. The cell group committed to actively migrating to form the prechordal mesendoderm should be carefully removed from the explant.

## 3 Results

### 3.1 Blocking cell division during gastrulation, but not at later stages, entails head and trunk defects

To investigate the role of cell division during the morphogenesis of the neural plate and dorsal mesoderm, we employed a combination of two DNA synthesis inhibitors: Hydroxyurea and Aphidicolin (HUA). This method of inhibiting cell division has already been demonstrated to be effective in *the Xenopus laevis* model (Harris and Hartenstein, 1991; Pokrovsky et al., 2021). First, we initiated the HUA treatment at stage 9.5 and stopped at stage 12.5. The treatment was confirmed effective, as embryos fixed at stage 13 exhibit many cell divisions when treated with DMSO but show almost none when treated with HUA (Figures 1D,E). We identified that blocking cell division during this developmental period does not prevent gastrulation and neurulation from occurring (Figures 1B,C), consistent with previous studies. It is worth noting, however, that 14 out of 17 HUA-treated embryos, revealed a process of neurulation that distinguishes from normal neurulation as the neural hinges are less prominent and the neural groove is shallower (compare Figure 1B with Figure 1C and Figure 1F with Figure 1G). Nevertheless, stage 20 HUA-treated formed notochord, paraxial mesoderm, and neural tube (Figures 1F,G).

Embryos treated with DMSO or HUA were systematically monitored during development and more than 90% (n = 34) of embryos exposed to HUA during gastrulation consistently developed pronounced anterior defects by stage 25 (compare Figure 2B’ with Figure 2C’). From a ventral view, it is clear how HUA-treated embryos show Figures 2D,G a narrow head when compared with DMSO-treated embryos. Additionally, HUA treated embryos elongate less than DMSO treated embryos (compare Figure 2B with Figure 2C). Later stages reveal severe abnormalities in HUA-treated embryos: underdeveloped head and truncated body (Figures 2E,E’,E”,F,F’,F”). Head defects and short trunk are also achieved when the HUA treatment is interrupted at stage 11.5 (Supplementary Figure S1). Which is consistent with the importance of cell division during gastrulation.

**FIGURE 3:**
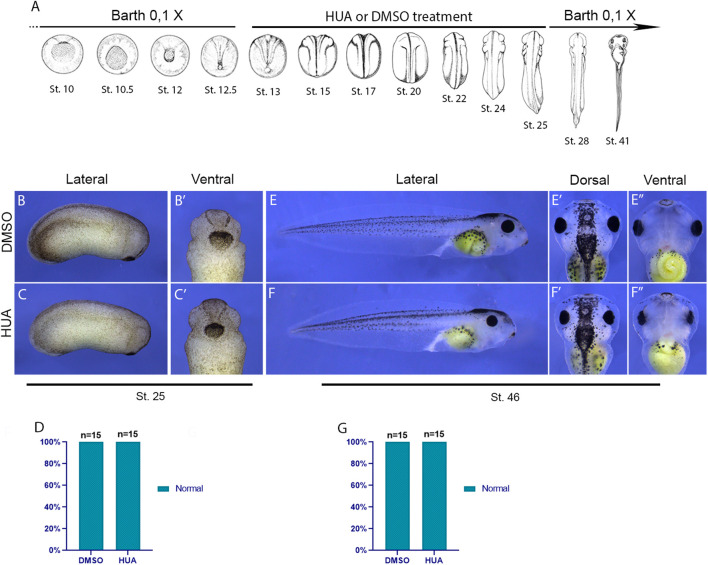
Blocking cell division during neurulation and tailbud stages does not cause head or trunk defects. **(A)** Embryos were kept in Barth’s 0.1X solution until stage 12.5, then transferred to HUA or DMSO solution, where they remained from stage 12.5 to stage 25, before being returned to Barth’s 0.1X solution. Wash off moment is indicated in red (sages illustrated according to Nieuwkoop and Faber, 1994). **(B)** Lateral view of a representative DMSO-treated embryo at stage 25. **(B′)** Ventral-anterior view of a representative DMSO-treated embryo at stage 25. **(C)** Lateral view of a representative HUA-treated embryo at stage 25. **(C′)** Ventral-anterior view of a representative HUA-treated embryo at stage 25. **(D)** Phenotype quantification at stage 25. **(E)** Lateral view of a representative DMSO-treated embryo at stage 46. **(E′)** Dorsal-anterior view of a representative DMSO-treated embryo at stage 46. **(E′′)** Ventral-anterior view of a representative DMSO-treated embryo at stage 46. **(F)** Lateral view of a representative HUA-treated embryo at stage 46. **(F′)** Dorsal-anterior view of a representative HUA-treated embryo at stage 46. **(F′′)** Ventral-anterior view of a representative HUA-treated embryo at stage 46. **(G)** Phenotype quantification at stage 46. (stages illustrated according to Nieuwkoop and Faber, 1994).

To determine whether the treatment is stage-specific, we questioned if blocking cell division after gastrulation to end, during neurulation and tailbud stages would also lead to malformations. Remarkably, blocking cell division between stages 13 and 25 did not affect the development of the head or trunk (Figure 3). Together, these findings demonstrate that while cell division is crucial for neural plate patterning during gastrulation, it becomes dispensable after neural tube closure or once anterior structures begin to form (Figure 3). Having established that the phenotype is stage specific, we chose stage 13 (the beginning of neurulation) as the ideal moment for a thorough investigation upon the patterning of the dorsal portion of the embryo.

**FIGURE 4:**
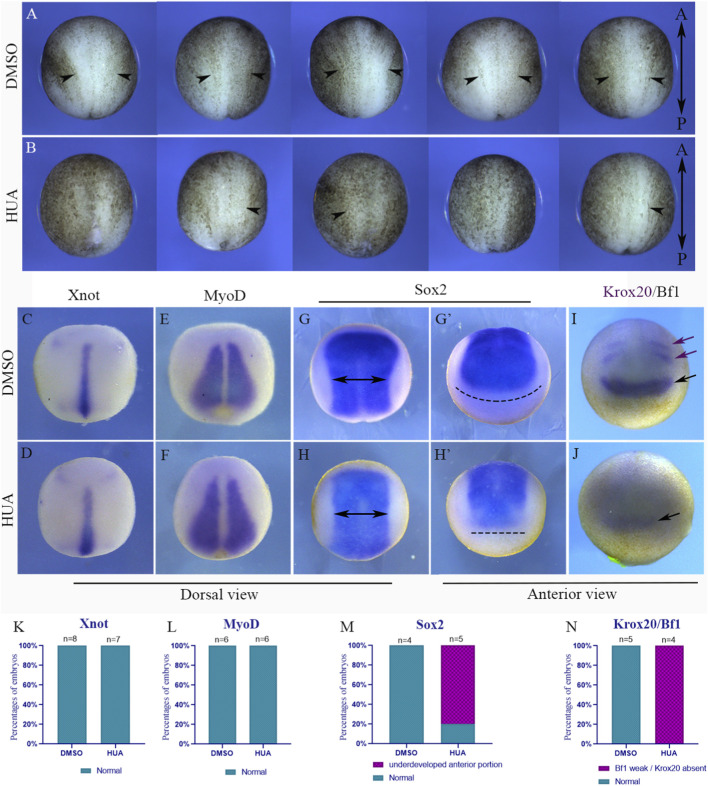
Blocking cell division during gastrulation affects neural plate shaping and patterning. **(A)** Dorsal view of five embryos at stage 13 treated with DMSO during gastrulation. **(B)** Dorsal view of five embryos at stage 13 treated with HUA during gastrulation. Arrowheads indicate the lateral hinges of the neural plate. Anterior is always at the top and posterior at the bottom. *In situ* hybridizations were conducted with embryos at stage 13 treated with DMSO **(C,E,G,G′,I)** or HUA **(D,F,H,H′,J)**. The embryos were marked for x*not* **(C,D)**, *myod* **(E,F)**, *Sox2* **(G,G′,H,H′)**, and krox20/bf1 **(I,J)**. Two head arrows in **(G)** and **(H)** indicate the *Sox2* expression domain width at the most dorsal portion of the embryo and are the same size. Dashed lines in **(G′)** and **(H′)** indicate the width of the most anterior region of the *sox2* domain in each condition. Purple arrows in **(I)** indicate Krox20 expression domains, while black arrows in **(I)** and **(J)** indicate the Bf1 domain. Quantification of each expression pattern is shown in **(K)** for *xnot*, in **(L)** for *myoD*, in **(M)** for *Sox2* and in **(N)** for *krox20/bf1*. All embryos (with the exceptions of **(G′,H′,I,J)**) are placed anterior up and posterior down.

To this purpose, we conducted a comparative analysis of the neural plate morphological patterns among various embryos treated with either dimethyl sulfoxide (DMSO) or HUA. Although each neural plate exhibits unique characteristics, the DMSO-treated embryos consistently demonstrate a coloration and morphological pattern indicative of healthy embryos: the neural plate region appears lighter than the surrounding ectoderm, and distinct neural folds extend from the anterior end to the blastopore (posterior pole) (Figure 4A). In contrast, embryos subjected to HUA treatment typically exhibit a significantly reduced area of light ectoderm and markedly discrete neural folds (compare Figures 4A,B).

Having established that HUA-treated embryos show significant differences in neural plate morphological patterning, we next asked if the embryonic territories were altered at stage 13. To address this, we selected marker genes of embryonic territories and performed *in situ* hybridizations. By comparing *sox2* domains in both DMSO and HUA embryos, it became evident that blocking cell division impacts the shaping of the neural plate, preventing the full development of its anterior portion (Figures 4G,G’,H,H’). Consequently, the anterior marker *bf1* expression is decreased and *kx20* expression is absent in 100% (n = 4) of the embryos (Figures 4I,J). This indicates that the forebrain, midbrain, and hindbrain domains are not properly patterned at this embryonic stage. On the other hand, the axial mesodermal marker *xnot* does not show relevant alterations when comparing HUA-treated embryos and DMSO-treated embryos (compare Figures 4C,D), indicating that notochord morphogenesis does not depend on cell division. Also, HUA-treated embryos show a normal pattern of *myoD* expression (Figures 4E,F), indicating that paraxial mesoderm morphogenesis also does not depend on cell division.

### 3.2 Cell divisions show strong A-P orientation across the ectoderm that is more pronounced in the dorsal area

Having established the importance of cell division for neuroectoderm patterning, we decided to track cell division and movement using transgenic nucleus-GFP embryos - lineage NXR_0139 Xla.Tg (CMV:hist2h2be-GFP; CMV:mRFP). Employing a low magnification at the light sheet microscope, we were able to detect involution, convergent extension, and epiboly occurring, while the neural plate is formed (Supplementary Video S1, Supplementary Figure S1). Remarkably, we could also observe a significant amount of cell division taking place in the ectoderm of the gastrulating embryo (Supplementary Video S1, Supplementary Figure S2). Previous cell tracking studies have shown the occurrence of cell division throughout the neural tube during neurulation (Christodoulou and Skourides, 2022). Here we focus on gastrulation and neurulation.

Aiming to determine whether those cell divisions exhibit any specific spatial-temporal pattern, we recorded gastrulation and neurulation with higher magnification and also considered three different areas of recording; in such a way that DNIMZ cells could be tracked alongside lateral non-involuting marginal zone (LNIMZ) and ventral non-involuting marginal zone (VNIMZ) (Supplementary Figures S3, S4 and Supplementary Videos S2, S3, S4).

**FIGURE 5:**
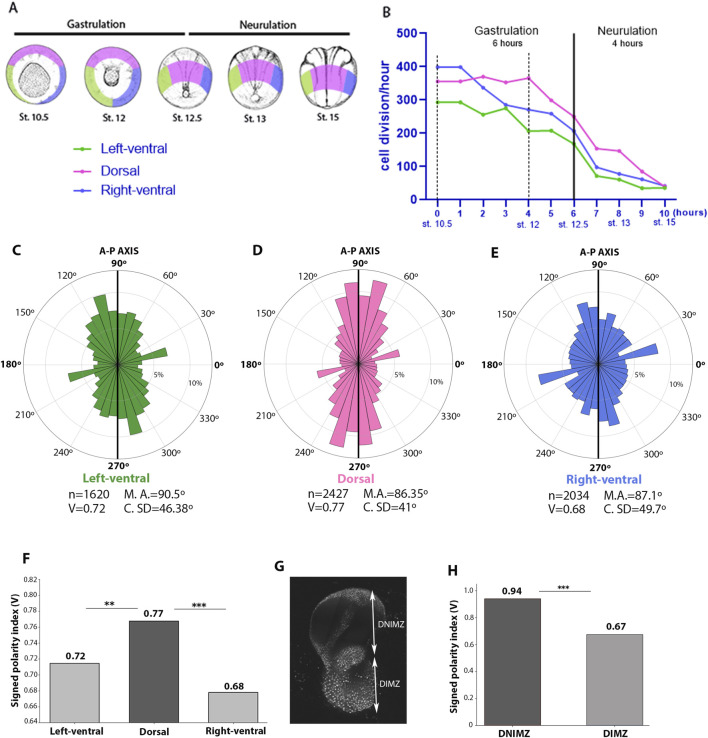
Mitotic cell divisions show strong anterior-posterior (A–P) orientation across the embryo’s ectoderm with higher strength at dorsal region. **(A)** The three distinct areas that were imaged are indicated in panel as the embryo goes throughout gastrulation and neurulation. Pink indicates the dorsal region; green denotes the left-ventral region; and blue represents the right-ventral region (sages illustrated according to Nieuwkoop and Faber, 1994). **(B)** The rate of cell divisions during the stages of gastrulation and neurulation, categorized by areas. **(C)** Rose plot showing the percentage (radial axis) of cell divisions in the left-ventral area that fall into each angular orientation. 90°–270° corresponds to the Anterior-Posterior (A–P) axis. **(D)** Rose plot showing the percentage (radial axis) of cell divisions in the dorsal area that fall into each angular orientation. **(E)** Rose plot showing the percentage (radial axis) of cell divisions in the right-ventral area that fall into each angular orientation. n: number of cell divisions; M.A.: Mean angle; v: Signed polarity index; **(C)**. SD: Circular standard deviation. **(F)** Comparison of the signed polarity index calculated for each area were made using two-tailed Welch’s t-test and Bonferroni correction. **(p < 0.01); ***(p < 0.001). The graphics from **(B–E)** reflect the three time-lapses performed from different angles of the same embryo exhibiting fluorescent nucleus-NXR_0139 Xla.Tg (CMV:hist2h2be-GFP; CMV:mRFP. **(G)** Keller explant performed from embryos with fluorescent nucleus-NXR_0139 Xla.Tg (CMV:hist2h2be-GFP; CMV:mRFP. Dorsal involuting marginal zone (DIMZ) and Dorsal non-involuting marginal zone (DNIMZ) are indicated. **(H)** Comparison of the signed polarity index calculated for each area. ***(p < 0.001). Other parameters: DIMZ (number of cell divisions = 54; mean angle = 56.2°; Circular standard deviation = 36.9°) and DNIMZ (number of cell divisions = 141; mean angle = 84.9°; Circular standard deviation = 19.5°).

All cell divisions were counted as described in the methodology section. Briefly, we identified cells passing from prophase to metaphase, anaphase and telophase in subsequential frames (usually 3-4 frames separated by 3 min each). By stablishing this pattern, based on nuclear morphology, we could determine the amount and direction of the cell divisions in each are recorded (Supplementary Figure S3). In total, 6,081 cell divisions were across the three different areas of the embryo and the rate of cell division decreases as gastrulation unfolds, taking a significant downward turn as neurulation begins (Figures 5A,B). This pattern is similar all around the marginal zone, although the rate of cell division at the dorsal area begins to decrease later than the others (at stage 12) (see Figures 5A,B).

The orientation angle of each cell division was measured and quantified as described in the methods. Mitotic divisions displayed strong anterior-posterior (A-P) orientation all across the embryo’s ectoderm (Figures 5C–E). In all cases, orientation strength was significant relative to the expected A-P axis of 90° (p < 0.0001, V-test). When comparing each region, however, we found out that A-P alignment strength at the dorsal area was significantly higher than at left-ventral area and the right-ventral area (dorsal vs. left-ventral: p = 0.0049; dorsal vs. right-ventral: p < 0.0001; see Figure 5F). On the other hand, left-ventral and right-ventral A-P alignment strength were not significant different (p = 0.152).

Notably, the dorsal area displayed the strongest A-P polarity combined with the largest number of mitotic divisions (Supplementary Figure S5). While both anterior and posterior dorsal halves exhibited more than 1,200 cell divisions, the posterior portion revealed a significant higher signed polarity index (0.83 vs. 0.72, p < 0.0001). Which indicates a dorsal-posterior bias regarding A-P oriented cell divisions across the embryo’s ectoderm.

We next examined whether this A-P oriented pattern of cell division is exclusive to the ectoderm or if it is also present in the dorsal involuting marginal zone (DIMZ). To address this, we imaged the development of a Keller ‘sandwich’ explant and quantified the orientation angle of each cell division using the same parameters as in the whole embryo (Figures 5G,H, Supplementary Video S5). During Keller explant development, DIMZ exhibited only 54 cell divisions, while dorsal non-involuting marginal zone (DNIMZ) exhibited 141 cell divisions (Figure 5H). As expected, DNIMZ showed a much higher A-P alignment strength than DIMZ (Figure 5H). These results align with the *in situ* analysis that show normal expression patterns of mesodermal markers and altered expression patterns of neural ectodermal markers (Figure 4).

## 4 Discussion

The results presented here demonstrate that cell division is abundant in the ectoderm of gastrulating embryos and tends to diminish as development progresses towards neurulation. Consistent with this, the inhibition of cell division during the early stages of gastrulation has a significant impact on developmental processes, whereas blocking cell division during the stages of neurulation and tailbud has a negligible effect. We demonstrated that inhibiting cell division during early gastrulation specifically impacts the shaping of the neural plate, which likely explains why anterior neural structures fail to develop properly thereafter.

In this context, our findings contribute for understanding the fundamental mechanisms through which brain malformations arise during development. A great number of studies has linked brain malformations to defects in neural tube closure (Curtin et al., 2003; Wood and Smith, 1984; Seller, 1995; Ravi et al., 2021; Frey and Hauser, 2003). However, it has also been reported that brain malformations can be caused by poorly specification of the forebrain, midbrain and hindbrain (ten Donkelaar et al., 2023; Parisi and Dobyns, 2003; Barkovich et al., 2012; Alkan et al., 2009; Niesen, 2002; Pombero et al., 2007; Sarnat and Flores‐Sarnat, 2004). The specification of those vesicles begins during neural plate morphogenesis, before neural tube closure takes place (Pombero et al., 2007; Martínez and Puelles, 2000; Cobos et al., 2001; Crossley et al., 2001; Foley et al., 2000; Kinder et al., 2001; Sasai and De Robertis, 1997; Weinstein and Hemmati-Brivanlou, 1997; Andoniadou and Martinez-Barbera, 2013). Importantly, several microcephaly genes identified (i.e., ASPM, BRIT1, Cep215, NDE1) are associated with centrosome and/or mitotic functions (Thornton and Woods, 2009; Nicholas et al., 2010; Bond et al., 2002; Feng and Walsh, 2004; Bakircioglu et al., 2011; Doobin et al., 2016). Our findings reinforce the importance of cell division in this context and also promote neural plate morphogenesis in *Xenopus laevis* as an appealing developmental window for one to challenge the cellular mechanisms underlying brain malformations.

Additionally, our living image results indicate that A-P alignment strength of cell divisions display a dorsal-posterior bias. Indeed, it has been proposed that the dorsal blastopore lip is a source of Wnt/planar cell polarity (PCP) signaling that patterns the neural plate (Mancini et al., 2021). Given that Wnt/PCP signaling pathway had already been demonstrate to influence cell division orientation (Davey and Moens, 2017; Wang and Nathans, 2007), it is expected that A-P cell division orientation is strongest at the dorsal-posterior ectoderm. Further experiments are needed in order to characterize this phenomenon and might shed light into an unresolved question related to the dorsal marginal zone lengthening during gastrulation: While it is true that the phenomenon of convergent extension (CE) has been thoroughly examined in *Xenopus laevis,* the reason why early radial intercalation results in anteroposterior (A-P) tissue elongation, rather than an overall spreading in all directions, remains to be elucidated (Wilson and Keller, 1991). In discussing this matter, Ray Keller suggests that radial intercalation leads to A-P tissue elongation probably due to an indiscernible level of medio-lateral intercalation or an unidentified mechanism (Wilson and Keller, 1991). Considering the findings presented here, we posit that the oriented cell division of the epithelial dorsal NIMZ is a good candidate on making the tissue to specifically flatten in the A-P direction. This will be a matter of future inquiries.

Our findings also indicate a limited occurrence of cell division at the dorsal involuting marginal zone (DIMZ) during gastrulation, which aligns with prior observations (Saka and Smith, 2001). Indeed, both axial and paraxial mesoderm were demonstrated to develop appropriately in the absence of cell division. We can conclude, from our results, that vertical signaling from the axial and paraxial mesoderm partially bypasses early failure in the A-P patterning of the neural plate. Importantly, however, the present study illuminates distinct phases of neural induction and their associated competencies. The initial phase involves the elongation of the neural plate in conjunction with anterior-posterior (A-P) patterning, which depends on cellular division. The subsequent phase being the neural tube closure, which is independent of cellular division. Notably, early abnormalities in neural plate patterning do not impair the subsequent neurulation phase; however, they become morphologically apparent at later stages, specifically during the tailbud and larval periods.

It is noteworthy that a significant evolutionary aspect is associated with the distinction between these two phases. While the formation of the neural tube is a mechanism that is specific to vertebrates, anterior-posterior (A-P) patterning and elongation of the neuroectoderm are embryologically connected to the deeply conserved phenomenon of A-P body axis formation. Indeed, it has been demonstrated that oriented cell division is fundamental for A-P body axis formation across a diverse array of animal species (Sander, 1976; Concha and Adams, 1998; Keller, 2006; Gong et al., 2004; Schoenwolf and Alvarez, 1989; Schoenwolf and Alvarez, 1992; Schoenwolf and Yuan, 1995; Wei and Mikawa, 2000). In this context, we would like to propose that neural plate elongation and patterning depend on A-P oriented cell division. Further investigation into the mechanisms responsible for neural plate elongation and patterning has the potential to provide significant insights into the fundamental evolutionary aspects of A-P body axis formation.

## Data Availability

The raw data supporting the conclusions of this article will be made available by the authors, without undue reservation.

## Supplementary material

SUPPLEMENTARY FIGURE 1 : Blocking cell division during gastrulation leads to head and trunk defects. **(A)** Embryos were treated with a combination of Hydroxyurea and Aphidicolin (HUA) or DMSO from stage 9.5 to stage 11.5 and then transferred to Barth’s 0.1X solution starting at stage 11.5. Wash off moment is indicated in red. **(B)** Lateral view of a representative DMSO-treated embryo at stage 22. **(B′)** Dorsal view of a representative DMSO-treated embryo at stage 22. **(C)** Lateral view of a representative HUA-treated embryo at stage 22. **(C′)** Dorsal view of a representative HUA-treated embryo at stage 22. **(D)** Lateral view of a representative DMSO-treated embryo at stage 28. **(D′)** Ventral-anterior view of a representative DMSO-treated embryo at stage 28. **(E)** Lateral view of a representative HUA-treated embryo at stage 28. **(E′)** Ventral-anterior view of a representative HUA-treated embryo at stage 28. **(F)** Phenotype quantification at stage 28.

SUPPLEMENTARY FIGURE 2 Methodology used to count cell divisions **(A)**. The three recording angles (green, pink and blue) were each divided into four quadrants. These quadrants were used to count the number of cell divisions and the orientation of these cell divisions during the gastrulation and neurulation process. **(B)** The three recording angles illustrated in the embryo at stage 11.5. **(C)** A 21 min period of the time lapse (8 frames out of 200) within the quadrant 7 showing 4 cell divisions taking place. The cell that is about to divide (single arrowheads) shows chromosome alignment before separation (coupled arrowheads). Metaphase is always observable before separation of the chromosomes (note nucleus morphology pointed by green arrowhead at 0 min; Orange arrowhead at 6 and 9 min; Pink arrowhead at 9 and 12 min; and Red arrowhead at 21 min). Cell division cannot be mistaken by radial intercalation, which is pointed by the blue arrow. In this case a cell from a deeper layer emerges to the more superficial layer without showing any sign of cell division (note how its signal is almost imperceptible at 15 min, than it becomes stronger at 18 min and even more at 21 min). The case of cell intercalation taking place close to a cell division was specifically chosen to show how those two kinds of events are easy to be distinguished. The images were made in a 20X increase.

SUPPLEMENTARY FIGURE 3 Development of the dorsal NIMZ during gastrulation and neurulation. Time-lapse of a transgenic Xenopus *laevis* embryo for fluorescent nucleus lineage NXR_0139 Xla.Tg (CMV:hist2h2be-GFP; CMV:mRFP, from stage 10.5 of development to stage 15 **(A–H)**. The approximate space of the DNIMZ is demarcated by a white hatched line. Red arrow indicates movement of cells that make up the DIMZ. White arrows indicate cell movement. Anterior pole (PA). Posterior pole (PP). Approximate border between NIMZ and IMZ (L1). Non-involuting dorsal marginal zone (DNIMZ). **(I)** *Xenopus laevis* embryo at the gastrula stage mounted in 0.5% agarose inside a glass tube that allows it to fit into the light sheet microscope. This assemblage can be rotated according to the observer’s needs so that the eyepiece, at the back, captures the required angle. The time-lapse was done in a 10X increase.

SUPPLEMENTARY FIGURE 4 Development of the dorsal, lateral and ventral marginal zone. Time-lapse of a transgenic Xenopus *laevis* embryo for fluorescent nucleus lineage NXR_0139 Xla.Tg(CMV:hist2h2be-GFP; CMV:mRFP, from stage 10.5 to stage 15 of development. One image was made from each angle indicated by the color pattern. Left lateral angle – ventral (green), dorsal angle (pink), right lateral angle – ventral (dark blue). The region imaged for each stage shown is indicated by the color pattern in the middle column (illustrations adapted from *xenbase.org*). An analysis performed by track-mate in ImageJ shows us the number of cells present in each of the angles chosen at the beginning of the experiment, and also the displacement route followed by each of these cells. The color pattern indicates the relative displacement of the cells. The red routes present a displacement 30 times greater than the dark blue routes. The images were made in a 20X increase.

SUPPLEMENTARY FIGURE 5 Anterior and posterior halves of each area recorded. **(A)** anterior left-ventral. **(B)** anterior dorsal. **(C)** anterior right-ventral. **(D)** posterior left-ventral. **(E)** posterior dorsal. **(F)** posterior right-ventral. In green, dorsal in pink and right ventral in blue). In each rose plot, radial axis indicates absolute number of cell divisions.90°–270° corresponds to anterior-posterior **(A–P)** body axis. n: number of cell divisions; M.A.: Mean angle; V: Signed polarity index; C. SD: Circular standard deviation.

SUPPLEMENTARY VIDEO 1 Whole embryo H2b – GFP. From stage 10.5 to stage 15. Dorsal view. Animal pole up, Vegetal pole down. ×10 magnification.

SUPPLEMENTARY VIDEO 2 Whole embryo H2b – GFP. Focus on dorsal NIMZ. From stage 10.5 to stage 15. Animal pole up, Vegetal pole down. ×20 magnification.

SUPPLEMENTARY VIDEO 3 Whole embryo H2b – GFP. Focus on left – ventral NIMZ. From stage 10.5 to stage 15. Animal pole up, Vegetal pole down. ×20 magnification.

SUPPLEMENTARY VIDEO 4 Whole embryo H2b – GFP. Focus on right – ventral NIMZ. From stage 10.5 to stage 15. Animal pole up, Vegetal pole down. ×20 magnification.

SUPPLEMENTARY VIDEO 5 Keller explant H2b – GFP. Fr on left – ventral NIMZ. From stage 10.5 to stage 15. Dorsal view. Animal pole up, Vegetal pole down. ×20 magnification.

## References

[B1] Abreu J. G. Ketpura N. I. Reversade B. De Robertis E. M. (2002). Connective-tissue growth factor (ctgf) modulates cell signalling by bmp and TGF-β. Nat. Cell Biol. 4 (8), 599–604. 10.1038/ncb826 12134160 PMC2387275

[B2] Alkan O. Kizilkilic O. Yildirim T. (2009). Malformations of the midbrain and hindbrain: a retrospective study and review of the literature. Cerebellum 8 (3), 355–365. 10.1007/s12311-009-0104-x 19337779

[B3] Andoniadou C. L. Martinez-Barbera J. P. (2013). Developmental mechanisms directing early anterior forebrain specification in vertebrates. Cell. Mol. Life Sci. 70, 3739–3752. 10.1007/s00018-013-1269-5 23397132 PMC3781296

[B4] Bakircioglu M. Carvalho O. P. Khurshid M. Cox J. J. Tuysuz B. Barak T. (2011). The essential role of centrosomal NDE1 in human cerebral cortex neurogenesis. Am. J. Hum. Genet. 88 (5), 523–535. 10.1016/j.ajhg.2011.03.019 21529752 PMC3146716

[B5] Barkovich A. J. Guerrini R. Kuzniecky R. I. Jackson G. D. Dobyns W. B. (2012). A developmental and genetic classification for malformations of cortical development: update 2012. Brain 135 (5), 1348–1369. 10.1093/brain/aws019 22427329 PMC3338922

[B6] Batschelet E. (1981). Circular statistics in biology.

[B7] Bond J. Roberts E. Mochida G. H. Hampshire D. J. Scott S. Askham J. M. (2002). ASPM is a major determinant of cerebral cortical size. Nat. Genet. 32 (2), 316–320. 10.1038/ng995 12355089

[B8] Brown J. M. Storey K. G. (2000). A region of the vertebrate neural plate in which neighbouring cells can adopt neural or epidermal fates. Curr. Biol. 10 (14), 869–872. 10.1016/s0960-9822(00)00601-1 10899008

[B9] Charrier J.-B. Teillet M.-A. Lapointe F. Le Douarin N. M. (1999). Defining subregions of Hensen’s node essential for caudalward movement, midline development and cell survival. Development 126 (21), 4771–4783. 10.1242/dev.126.21.4771 10518494

[B10] Chartrain I. Page Y. L. Hatte G. Ko R. Kubiak J. Z. Tassan J. (2011). Cell-cycle dependent localization of MELK and its new partner RACK1 in epithelial *versus* mesenchyme-like cells in Xenopus embryo. Biol Open 2(10):1037–1048. 10.1242/bio.20136080 PMC3798187 24167714

[B11] Christodoulou N. Skourides P. A. (2022). Distinct spatiotemporal contribution of morphogenetic events and mechanical tissue coupling during Xenopus neural tube closure. Development 149 (13), dev200358. 10.1242/dev.200358 35662330 PMC9340557

[B12] Cobos I. Shimamura K. Rubenstein J. L. R. Martínez S. Puelles L. (2001). Fate map of the avian anterior forebrain at the four-somite stage, based on the analysis of quail–chick chimeras. Dev. Biol. 239 (1), 46–67. 10.1006/dbio.2001.0423 11784018

[B13] Concha M. L. Adams R. J. (1998). Oriented cell divisions and cellular morphogenesis in the zebrafish gastrula and neurula: a time-lapse analysis. Development 125 (6), 983–994. 10.1242/dev.125.6.983 9463345

[B14] Crossley P. H. Martinez S. Ohkubo Y. Rubenstein J. L. R. (2001). Coordinate expression of Fgf8, Otx2, Bmp4, and Shh in the rostral prosencephalon during development of the telencephalic and optic vesicles. Neuroscience 108 (2), 183–206. 10.1016/s0306-4522(01)00411-0 11734354

[B15] Curtin J. A. Quint E. Tsipouri V. Arkell R. M. Cattanach B. Copp A. J. (2003). Mutation of Celsr1 disrupts planar polarity of inner ear hair cells and causes severe neural tube defects in the mouse. Curr. Biol. 13 (13), 1129–1133. 10.1016/s0960-9822(03)00374-9 12842012

[B16] Davey C. F. Moens C. B. (2017). Planar cell polarity in moving cells: think globally, act locally. Dev 144 (2), 187–200. 10.1242/dev.122804 28096212 PMC5394761

[B17] Doniach T. Phillips C. R. Gerhart J. C. (1992). Planar induction of anteroposterior pattern in the developing central nervous system of *Xenopus laevis* . Science 257 (5069), 542–545. 10.1126/science.1636091 1636091

[B18] Doobin D. J. Kemal S. Dantas T. J. Vallee R. B. (2016). Severe NDE1-mediated microcephaly results from neural progenitor cell cycle arrests at multiple specific stages. Nat. Commun. 7 (1), 12551. 10.1038/ncomms12551 27553190 PMC4999518

[B19] Feng Y. Walsh C. A. (2004). Mitotic spindle regulation by Nde1 controls cerebral cortical size. Neuron 44 (2), 279–293. 10.1016/j.neuron.2004.09.023 15473967

[B20] Foley A. C. Skromne I. Stern C. D. (2000). Reconciling different models of forebrain induction and patterning: a dual role for the hypoblast. Development 127 (17), 3839–3854. 10.1242/dev.127.17.3839 10934028

[B21] Frey L. Hauser W. A. (2003). Epidemiology of neural tube defects. Epilepsia 44, 4–13. 10.1046/j.1528-1157.44.s3.2.x 12790881

[B22] Giese W. Albrecht J. P. Oppenheim O. Akmeriç E. B. Kraxner J. Schmidt D. (2025). Polarity-JaM: an image analysis toolbox for cell polarity, junction and morphology quantification. Nat. Commun. 16 (1), 1474. 10.1038/s41467-025-56643-x 39922822 PMC11807127

[B23] Gilbert S. F. Barresi M. J. F. (2016). Dev. Biology, 810. 10.1002/ajmg.a.38166

[B24] Gong Y. Mo C. Fraser S. E. (2004). Planar cell polarity signalling controls cell division orientation during zebrafish gastrulation. Nature 430 (7000), 689–693. 10.1038/nature02796 15254551

[B25] Harris W. A. Hartenstein V. (1991). Neuronal determination without cell division in xenopus embryos. Neuron 6 (4), 499–515. 10.1016/0896-6273(91)90053-3 1901716

[B26] Hatada Y. Stern C. D. (1994). A fate map of the epiblast of the early chick embryo. Development 120 (10), 2879–2889. 10.1242/dev.120.10.2879 7607078

[B27] Hatte G. Tramier M. Prigent C. Tassan J. (2014). Epithelial cell division in the *Xenopus laevis* embryo during gastrulation. Int. J. Dev. Biol. 781 (December), 775–781. 10.1387/ijdb.140277jt 26154319

[B28] Keller R. E. (1978). Time‐lapse cinemicrographic analysis of superficial cell behavior during and prior to gastrulation in *Xenopus laevis* . J. Morphol. 157 (2), 223–247. 10.1002/jmor.1051570209 30235909

[B29] Keller R. E. Danilchik M. Gimlich R. Shih J. (1985). The function and mechanism of convergent extension during gastrulation of Xenopus laevis. J. Embryol. exp. Morph. 89, Supplement, 185–209. 10.1242/dev.89.supplement.185 3831213

[B30] Keller R. (2006). Mechanisms of elongation in embryogenesis. Development 133 (12), 2291–2302. 10.1242/dev.02406 16720874

[B31] Keller R. A. Y. Danilchik M. (1988). Regional expression, pattern and timing of convergence and extension during gastrulation of *Xenopus laevis* . Development 103, 193–209. 10.1242/dev.103.1.193 3197629

[B32] Keller R. Tibbetts P. (1989). Mediolateral cell intercalation in the dorsal, axial mesoderm of *Xenopus laevis* . Dev. Biol. 131 (2), 539–549. 10.1016/s0012-1606(89)80024-7 2463948

[B33] Keller R. Shih J. Sater A. (1992a). The cellular basis of the convergence and extension of the Xenopus neural plate. Dev. Dyn. 193 (3), 199–217. 10.1002/aja.1001930302 1600240

[B34] Keller R. Shih J. Sater A. K. Moreno C. (1992b). Planar induction of convergence and extension of the neural plate by the organizer of Xenopus. Dev. Dyn. 193 (3), 218–234. 10.1002/aja.1001930303 1600241

[B35] Kinder S. J. Tsang T. E. Ang S.-L. Behringer R. R. Tam P. P. L. (2001). Defects of the body plan of mutant embryos lacking Lim1, Otx2 or Hnf3 beta activity. Int. J. Dev. Biol. 45 (1), 347–356. 11291865

[B36] Le Douarin N. M. Halpern M. E. (2000). Discussion point: origin and specification of the neural tube floor plate: insights from the chick and zebrafish. Curr. Opin. Neurobiol. 10 (1), 23–30. 10.1016/s0959-4388(99)00062-8 10679443

[B37] Lee C. Kieserman E. Gray R. S. Park T. J. Wallingford J. (2008). Whole-mount fluorescence immunocytochemistry on Xenopus embryos. Cold Spring Harb. Protoc. 2008 (2), pdb–prot4957. 10.1101/pdb.prot4957 21356778

[B38] López S. L. Paganelli A. R. Rosato Siri M. V. Ocaña O. H. Franco P. G. Carrasco A. E. (2003). Notch activates sonic hedgehog and both are involved in the specification of dorsal midline cell-fates in Xenopus. Development 130 (10), 2225–2238. 10.1242/dev.00443 12668635

[B39] López S. L. Rosato-Siri M. V. Franco P. G. Paganelli A. R. Carrasco A. E. (2005). The Notch-target gene hairy2a impedes the involution of notochordal cells by promoting floor plate fates in Xenopus embryos. Development 132 (5), 1035 LP–1046. 10.1242/dev.01659 15689375

[B40] Mancini P. Ossipova O. Sokol S. Y. (2021). The dorsal blastopore lip is a source of signals inducing planar cell polarity in the Xenopus neural plate. Biol. Open 10 (7), bio058761. 10.1242/bio.058761 34259326 PMC8325942

[B41] Martínez S. Puelles L. (2000). Neurogenetic compartments of the mouse diencephalon and some characteristic gene expression patterns. Mouse Brain Dev. 30, 91–106. 10.1007/978-3-540-48002-0_4 10857186

[B42] Nicholas A. K. Khurshid M. Désir J. Carvalho O. P. Cox J. J. Thornton G. (2010). WDR62 is associated with the spindle pole and is mutated in human microcephaly. Nat. Genet. 42 (11), 1010–1014. 10.1038/ng.682 20890279 PMC5605390

[B43] Niesen C. E. (2002). Malformations of the posterior fossa: current perspectives. Semin. Pediatr. Neurol. 9 (4), 320–334. 10.1053/spen.2002.32508 12523556

[B44] Nieuwkoop P. D. Faber J. (1994). Normal table of *Xenopus laevis* (Daudin): a systematical and chronological survey of the development from the fertilized egg till the end of metamorphosis.

[B45] Parisi M. A. Dobyns W. B. (2003). Human malformations of the midbrain and hindbrain: review and proposed classification scheme. Mol. Genet. Metab. 80 (1), 36–53. 10.1016/j.ymgme.2003.08.010 14567956

[B60] Pokrovsky D. Forné I. Straub T. Imhof A. Rupp R. A. W. (2021). A systemic cell cycle block impacts stage-specific histone modification profiles during Xenopus embryogenesis. PLoS Biol. 19 (9), e3001377. 10.1371/journal.pbio.3001377 34491983 PMC8535184

[B46] Pombero A. Valdes L. Vieira C. Martinez S. (2007). Developmental mechanisms and experimental models to understand forebrain malformative diseases. Genes. Brain Behav. 6, 45–52. 10.1111/j.1601-183X.2007.00322.x 17543039

[B47] Ravi K. S. Hassan S. B. Pasi R. Mittra S. Kumar R. (2021). Neural tube defects: different types and brief review of neurulation process and its clinical implication. J. Fam. Med. Prim. Care 10 (12), 4383–4390. 10.4103/jfmpc.jfmpc_904_21 PMC8884297 35280642

[B48] Reversade B. De Robertis E. M. (2005). Regulation of ADMP and BMP2/4/7 at opposite embryonic poles generates a self-regulating morphogenetic field. Cell 123 (6), 1147–1160. 10.1016/j.cell.2005.08.047 16360041 PMC2292129

[B49] Saka Y. Smith J. C. (2001). Spatial and temporal patterns of cell division during early Xenopus embryogenesis. Dev. Biol. 229 (2), 307–318. 10.1006/dbio.2000.0101 11150237

[B50] Sander K. (1976). Specification of the basic body pattern in insect embryogenesis. Adv Insect Phys 12 (C), 125–238. 10.1016/S0065-2806(08)60255-6

[B51] Sarnat H. B. Flores‐Sarnat L. (2004). Integrative classification of morphology and molecular genetics in central nervous system malformations. Am. J. Med. Genet. Part A 126 (4), 386–392. 10.1002/ajmg.a.20663 15098236

[B52] Sasai Y. De Robertis E. M. (1997). Ectodermal patterning in vertebrate embryos. Dev. Biol. 182 (1), 5–20. 10.1006/dbio.1996.8445 9073437

[B53] Schoenwolf G. C. Alvarez I. S. (1989). Roles of neuroepithelial cell rearrangement and division in shaping of the avian neural plate. Development 106 (3), 427–439. 10.1242/dev.106.3.427 2598817

[B54] Schoenwolf G. C. Alvarez I. S. (1992). Role of cell rearrangement in axial morphogenesis. Curr. Top. Dev. Biol. 27 (C), 129–173. 10.1016/s0070-2153(08)60534-7 1424762

[B55] Schoenwolf G. C. Yuan S. (1995). Experimental analyses of the rearrangement of ectodermal cells during gastrulation and neurulation in avian embryos. Cell Tissue Res. 280 (2), 243–251. 10.1007/BF00307795 7781022

[B56] Selleck M. A. J. Stern C. D. (1991). Fate mapping and cell lineage analysis of Hensen’s node in the chick embryo. Development 112 (2), 615–626. 10.1242/dev.112.2.615 1794328

[B57] Seller M. J. (1995). Sex, neural tube defects, and multisite closure of the human neural tube. Am. J. Med. Genet. 58 (4), 332–336. 10.1002/ajmg.1320580406 8533841

[B58] Sive H. L. Grainger R. M. Harland R. (2000). Early development of Xenopus laevis: a laboratory manuale. Cold Spring Harbor, NY: Cold Srping Harbor Laboratory Press (CSHL).

[B59] Steventon B. Arias A. M. (2017). Evo-engineering and the cellular and molecular origins of the vertebrate spinal cord. Dev. Biol. 432 (1), 3–13. 10.1016/j.ydbio.2017.01.021 28192080

[B61] ten Donkelaar H. J. Lammens M. Cruysberg J. R. M. Ulzen K. K. Hori A. Shiota K. (2023). “Development and developmental disorders of the forebrain,” in Clinical neuroembryology: development and developmental disorders of the human central nervous system (Cham: Springer International Publishing), 595–724. 10.1007/978-3-031-26098-8_9

[B62] Thornton G. K. Woods C. G. (2009). Primary microcephaly: do all roads lead to Rome? Trends Genet. 25 (11), 501–510. 10.1016/j.tig.2009.09.011 19850369 PMC2816178

[B91] Velloso I. Araujo R. Horb M. Abreu J. G. (2025). Cell division during Xenopus gastrulation influences neuroectoderm patterning. bioRxiv 2025.11.11.687238. 10.1101/2025.11.11.687238 PMC13144160 42099383

[B63] Wang Y. Nathans J. (2007). Tissue/planar cell polarity in vertebrates: new insights and new questions. Development 134 (4), 647–658. 10.1242/dev.02772 17259302

[B64] Wei Y. Mikawa T. (2000). Formation of the avian primitive streak from spatially restricted blastoderm: evidence for polarized cell division in the elongating streak. Development 127 (1), 87–96. 10.1242/dev.127.1.87 10654603

[B65] Weinstein D. C. Hemmati-Brivanlou A. (1997). Neural induction in Xenopus laevis: evidence for the default model. Curr. Opin. Neurobiol. 7 (1), 7–12. 10.1016/s0959-4388(97)80114-6 9039789

[B66] Wilson P. Keller R. (1991). Cell rearrangement during gastrulation of Xenopuis: direct obseravtion of cultured explants. Development 112, 289–300. 10.1242/dev.112.1.289 1769334

[B67] Wood L. R. Smith M. T. (1984). Generation of anencephaly: 1. Aberrant neurulation and 2. Conversion of exencephaly to anencephaly. J. Neuropathol. Exp. Neurol. 43 (6), 620–633. 6502191

