# Supplementary figures and images for: Cell division during Xenopus gastrulation influences neuroectoderm patterning
Source: Front Cell Dev Biol. 2026 Apr 22;14:1798565. doi: 10.3389/fcell.2026.1798565 (PMC13144160; doi:10.3389/fcell.2026.1798565)

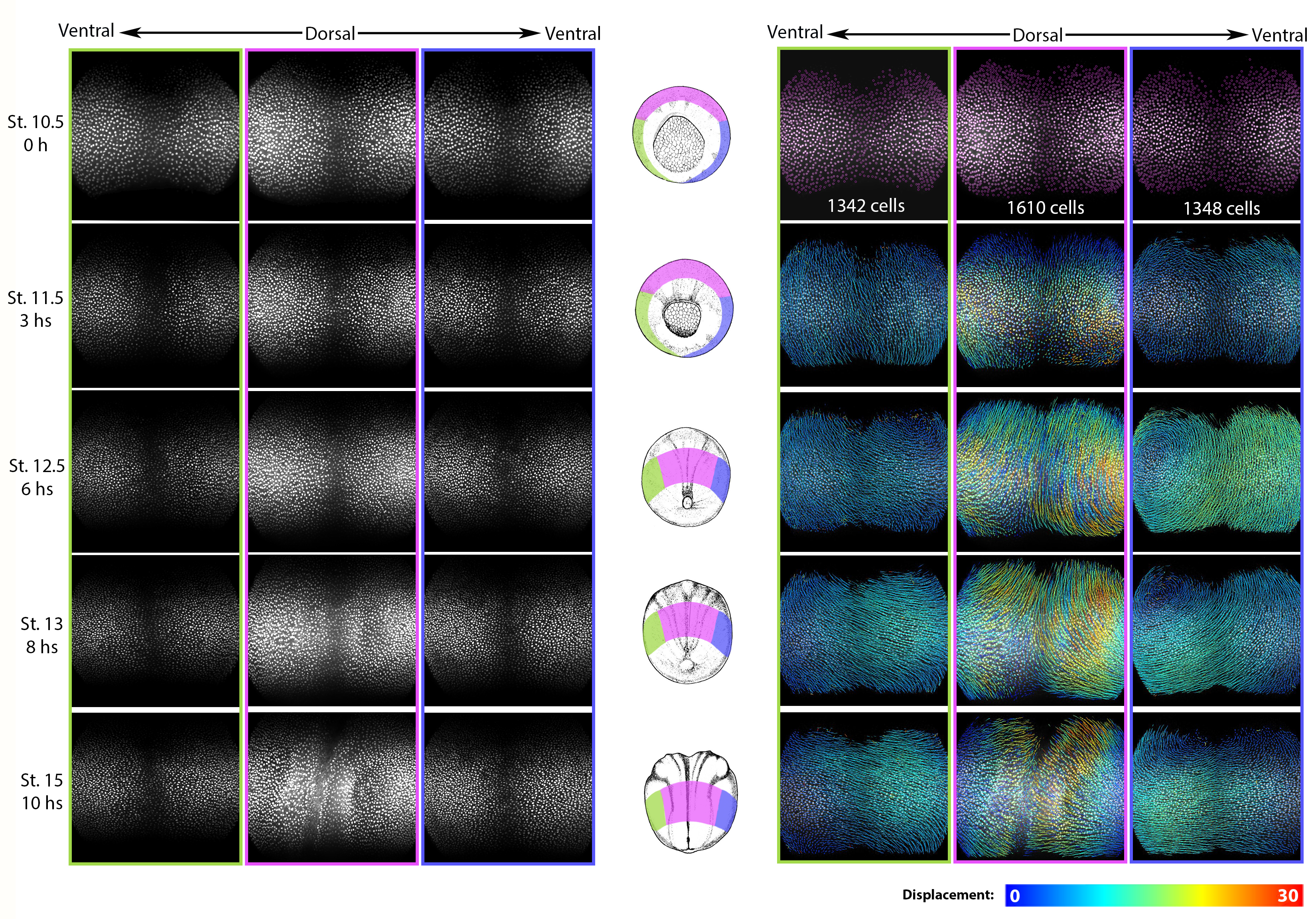

Supplement: Supplementary file 2 [file Image3.tif]

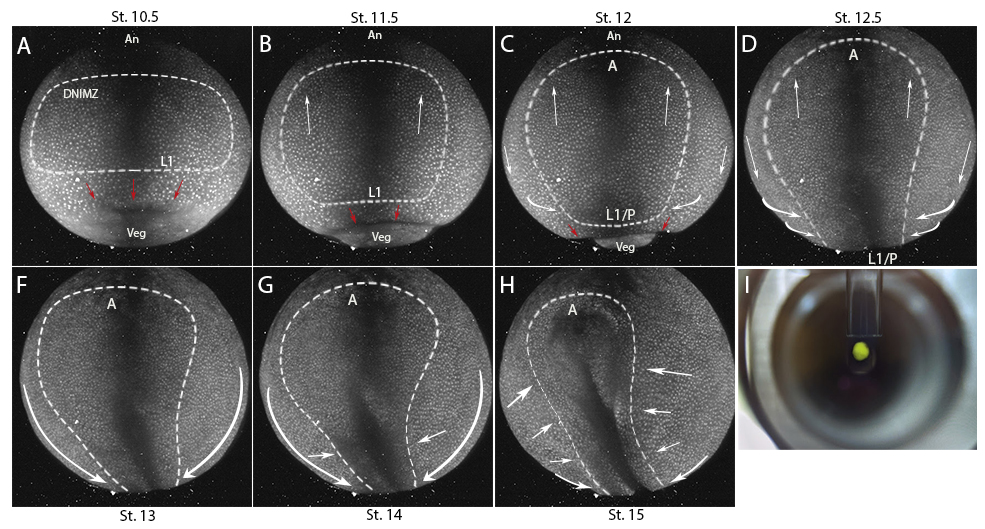

Supplement: Supplementary file 3 [file Image4.tif]

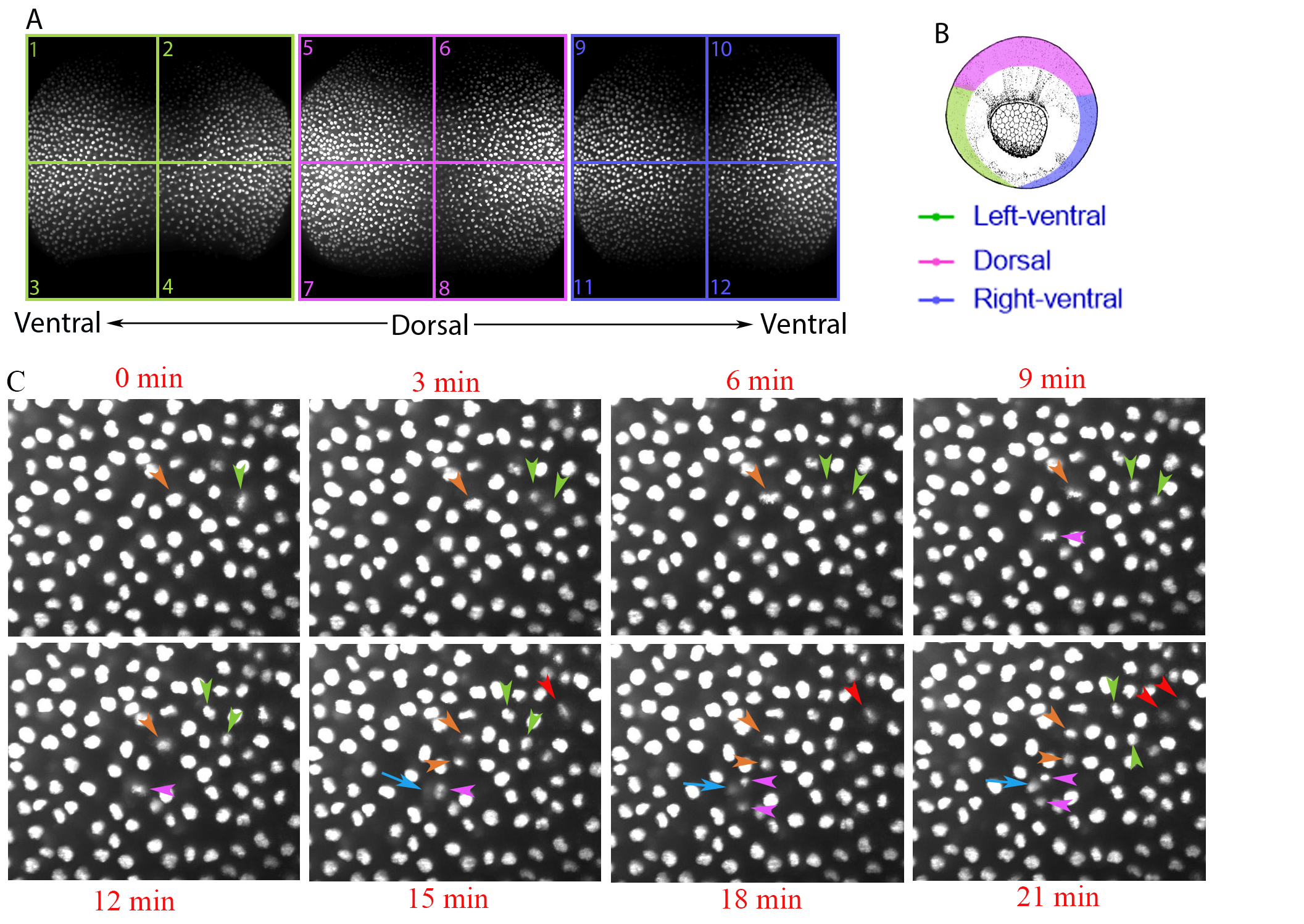

Supplement: Supplementary file 4 [file Image2.tif]

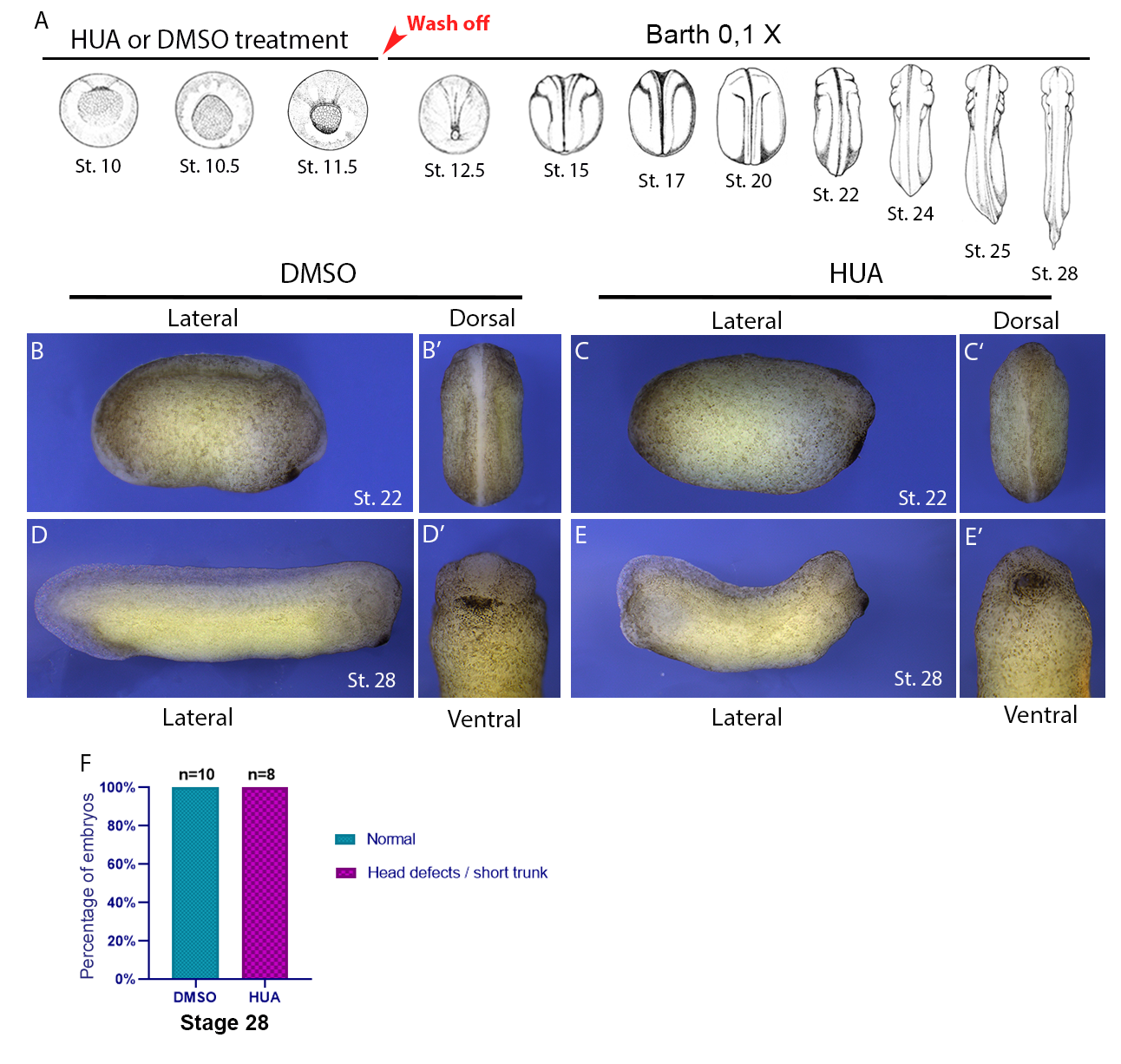

Supplement: Supplementary file 5 [file Image1.tif]

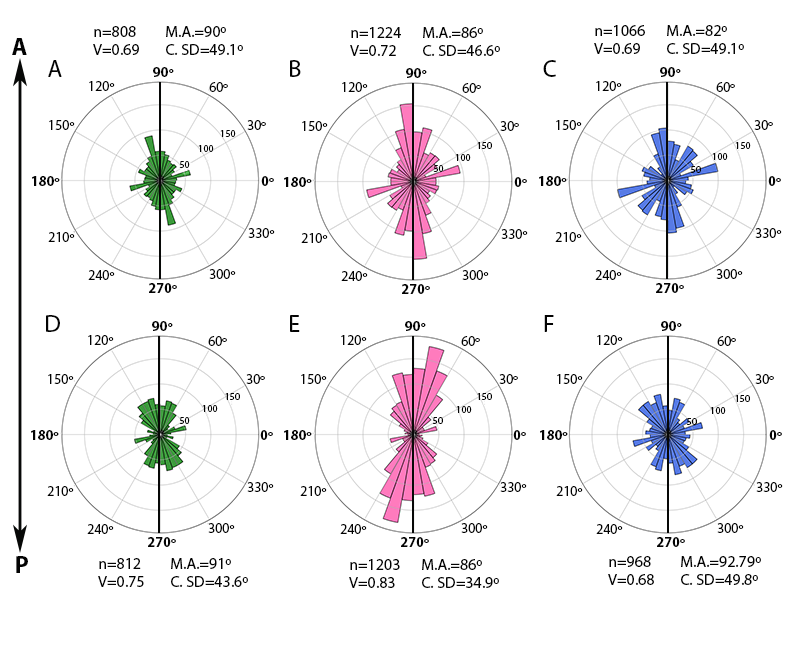

Supplement: Supplementary file 8 [file Image5.tif]
